# FRAX: re-adjust or re-think

**DOI:** 10.1007/s11657-020-00827-z

**Published:** 2020-09-28

**Authors:** Yasser El Miedany

**Affiliations:** King’s College London, London, England

**Keywords:** Osteoporosis, FRAX, Fracture probability, Clinical risk factors, Intervention thresholds, Risk assessment, Screening, BMD, Adjustment, Artificial intelligence

## Abstract

Since its development in 2008, FRAX has booked its place in the standard day to day management of osteoporosis. The FRAX tool has been appreciated for its simplicity and applicability for use in primary care, but criticised for the same reason, as it does not take into account exposure response. To address some of these limitations, relatively simple arithmetic procedures have been proposed to be applied to the conventional FRAX estimates of hip and major fracture probabilities aiming at adjustment of the probability assessment. However, as the list of these adjustments got longer, this has reflected on its implementation in the standard practice and gave FRAX a patchy look. Consequently, raises the need to re-think of the current FRAX and whether a second generation of the tool is required to address the perceived limitations of the original FRAX. This article will discuss both point of views of re-adjustment and re-thinking.

## Introduction

The principle aim of osteoporosis treatment has been preventing or decreasing the risk of fragility fractures; therefore, a critical factor for patients’ management is the ability to assess fracture risk, identifying those eligible for intervention [1, 2]. The World Health Organization (WHO) Collaborating Centre at Sheffield, UK, released FRAX in 2008—a computer-based algorithm (http://www.shef.ac.uk/FRAX) that calculates individualised 10-year probability of hip and major osteoporotic fracture (clinical spine, distal forearm, and proximal humerus). As the probability of fractures differ considerably within and across different world regions [3, 4], FRAX models had to be calibrated to the fracture and death epidemiology in individual countries. At the time when FRAX was launched, models were only available for 8 nations. Currently, 71 models are available for 66 countries comprising more than 80% of the world population [5]. FRAX is available in 35 languages and approximately 3 million visits are received on the FRAX website annually. In 2018, the FRAX tool celebrated its 10th birthday [6].

FRAX tool is made up of seven dichotomous clinical risk factors which include prior fragility fracture, parental hip fracture, smoking, systemic glucocorticoid use, excess alcohol intake, rheumatoid arthritis, and other causes of secondary osteoporosis. In addition to age and sex and body mass index (BMI), these risk factors contribute to estimating a 10-year fracture probability, independent of bone mineral density (BMD). However, BMD at the femoral neck is an optional input variable [6, 7]. Earlier data had revealed that the sensitivity of BMD measurements for fracture prediction is low; therefore, FRAX represented a conceptual, clinical development, which superseded management previously purely based on BMD T-score [8, 9]. Furthermore, under a competing mortality framework, FRAX provides robust fracture prediction and calibration [10].

Similar to the experience encountered when the definition of osteoporosis was proposed, the introduction of FRAX engendered some controversy. End-user, doctors, and allied health care professionals commended the tool for its simplicity, whereas it was criticised by academics for the same reason. It has become evident over the past 10 years, like all available clinical risk assessment tools, FRAX has several limitations which need to be considered when the results are interpreted. Kanis et al. [9] described these limitations as “Teething troubles”. Recent reviews of FRAX limitations [11] revealed variable critical points which include the fact that FRAX assessment does not take into account dose-responses for several risk factors. Another important limitation of FRAX, originally intended to identify high fracture risk, is the impact of prior fracture on the calculated absolute fracture risk. Clinicians know that whilst all fractures are important, not all fractures are equal; however, FRAX only accepts a binary input for previous fracture [12]. Some examples are that two prior fractures carry a much higher risk than a single prior fracture [13]. Also, fractures which involve the hip and spine carry a higher risk for recurrent fracture than fractures of the distal extremities. A further example is that, a vertebral fracture with a small residual deformity is a weaker prognostic risk factor than a more severe deformity [14]. Other factors are that dose-responses are also evident for glucocorticoid exposure [15], smoking cigarette [16], and alcohol intake [17]. Lack of provision for lumbar spine BMD which is commonly recommended in treatment guidelines and the absence of measurements of the material or bone structural properties are further concerns. More recently, the time dependence of fracture risk assessment has attracted attention, and studies have revealed that there is a period of imminent risk following a fracture event [18, 19]. This creates an opportunity for early intervention, with potentially more potent agents, which could reverse that risk [20].

As these different scenarios have not all been accommodated within the FRAX algorithm, such limitations have tempered clinical judgement. To address these limitations, relatively simple arithmetic adjustments have been proposed, which can be applied to the conventional FRAX estimates of probabilities of hip/major osteoporosis fracture to adjust the probability assessment [21].

## FRAX: to re-adjust

Some guidance concerning the quantum of the medications impact, whether type of medication or its dose, or fracture recency time, have been suggested to aid the patients’ assessment, given the binary input of the seven clinical risk factors included in the FRAX tool. The guidance was later expanded to accommodate other clinical risk factors. Table 1 shows a list of these proposed adjustments to FRAX.

**Table 1:** A list of these proposed adjustments to FRAX

Clinical risk factor	Reference
Recency of vertebral fracture	[18]
Glucocorticoids dose	[22]
Concurrent data on lumbar spine BMD	[23, 24]
Trabecular bone score	[25–28]
Hip axis length	[29]
Falls history	[30]
Immigration status	[31]
Type 2 diabetes	[32, 33]
Androgen depletion/hormone antagonist therapy	[34]
Chronic kidney disease	[35]

## FRAX adjustment to the recency of fractures

Most recently, FRAX adjustment has considered the recency of vertebral fracture(s) as a risk for fracture. This was based on the substantial evidence indicating that the risk of a subsequent osteoporotic fracture is rather acute immediately after the index fracture, and that this wanes progressively with time [36–39]. Therefore, the incidence of second fracture is particularly high in the first 2 years after the index event [40]. As the FRAX tool provides fracture probabilities associated with a prior fracture, irrespective of its recency, this consequently underestimates fracture probability where the prior fracture occurred within 2 years. Adjustments have been proposed to FRAX calculation for a recent vertebral fracture. For example, for a woman at age 70 years, a prior clinical vertebral fracture within the past 2 years is associated with a 1.52-fold higher fracture probability than for a woman of the same age with a prior fragility fracture of uncertain recency [39] (Table 2). So, for example, a recent clinical vertebral fracture raises the fracture probability from 16 to 24%. Depending on the age, adjustments ratios range from 1.04 to 2.47. Adjustment ratios for recent fractures at other sites have yet to be determined. Table 2 shows 10-year probability of major osteoporotic fracture (MOF) for Icelandic women at different ages, categorised by (A) a clinical vertebral fracture within the previous 2 years and (B) a prior fracture of undetermined recency [41].

**Table 2:** Ten-year probability of major osteoporotic fracture (MOF) for Icelandic women at different ages, categorised by (A) a clinical vertebral fracture within the previous 2 years and (B) a prior fracture of undetermined recency. Data from Kanis et al. 2020 [41]

Age	10-year probability of MOF		Ratio
	(A) Recent vertebral fracture	(B) Prior fracture in adult life	
50	29.0	11.7	2.47
60	36.1	19.4	1.86
70	41.9	27.6	1.52
80	42.5	34.2	1.·24
90	34.7	33.3	1.04

BMI set at 25 kg/m^2^

The right-hand column provides the ratio by which to adjust FRAX probabilities by virtue of a recent clinical vertebral fracture. Probabilities and ratios are derived from the UK

## FRAX adjustment using trabecular bone score

Trabecular bone score (TBS), a texture-based measurement derived from spine DXA images, has attracted clinical interest due to its proven ability to predict fracture risk independent of BMD and FRAX score. It has been recently reported that TBS can be used to adjust the FRAX score, with the aim of improving fracture risk prediction. In particular, this is useful where guidelines recommend the use of FRAX in order to determine the initiation of osteoporosis therapy. Where the osteoporosis diagnosis and treatment are primarily based on BMD T-score, a risk-equivalent T-score can be calculated based upon lumbar spine TBS [42]. Using data from a clinical registry of 45,185 women aged 40 years and older with mean follow-up 7.4 years to assess the incidence of major osteoporosis fracture(s) (MOF) (*N* = 3925), it was possible to create models to derive a TBS offset to the BMD T-score that would give the same risk as a unit change in BMD T-score for the femur neck, total hip, and lumbar spine [43]. By using the TBS-adjusted BMD T-score versus the unadjusted BMD T-score, risk stratification and model fit were improved. Using this approach was equivalent to the existing TBS-adjustment to FRAX [42].

Using the same large cohort, Martineau et al. [44] examined the incremental value of lumbar spine TBS on fracture risk assessment in relation to baseline characteristics: age, sex, BMI, prior fracture, rheumatoid arthritis (RA), glucocorticoid use, femoral neck T-score, chronic obstructive lung disease, high alcohol use, number of comorbidities, diabetes, secondary osteoporosis, and osteoporosis treatment. This found that TBS was sensitive to the effects of multiple risk factors for fracture. Also, TBS improved fracture risk assessment in multiple subgroups. The largest gradient of risk (HR per SD reduction) for fracture prediction with TBS was seen for age less than 65 versus 65+ (MOF *P*-interaction = 0.004, hip fracture *P*-interaction < 0.001), without versus with prior fracture (MOF *P*-interaction = 0.003, hip fracture *P*-interaction 0.048), without versus with glucocorticoid use (HF P-interaction 0.029), lower versus higher comorbidity score (HF *P*-interaction < 0.001), and without versus with osteoporosis treatment (MOF *P*-interaction = 0.005).

## FRAX adjustment to the glucocorticoids dose

A further limitation of FRAX is that use of oral glucocorticoids is recorded as a dichotomous risk factor, not taking into account the dose or the duration of glucocorticoids use; or taking into account the difference in the risk between prior and current glucocorticoids use [34]. FRAX postulates an average dose of prednisolone of 2.5–7.5 mg/day or its equivalent which could underestimate fracture risk in patients taking higher doses and overestimate risk in those taking lower doses. Although the highest risk in glucocorticoids users is vertebral fractures, FRAX predicted value has mainly been validated for non-vertebral fractures. Adjustment of FRAX has also been proposed for both men and postmenopausal women aged 50 years old or over with lower or higher doses than 2.5–7.5 mg/day [22]. Based on this proposal, looking at UK data, dose adjustments have been made. In patients taking low doses of glucocorticoids (< 2.5 mg/day), the probability of a major osteoporotic fracture can be decreased by about 20% and hip fracture by about 35%, depending on age, whereas the respective probabilities can be increased by about 15% and 20% for doses > 7.5 mg daily (Table 3). As glucocorticoid use may be associated with more marked BMD reductions at the spine than at the femoral neck, adjustments can also be made for marked discordance in T-scores between these sites [23].

**Table 3:** Adjustment of FRAX 10-year probability of fracture adjusted for the glucocorticoid dose

Type of fracture risk	Current daily glucocorticoid dose	Prednisolone equivalent (mg per day)	Correcting factor*
Major osteoporotic fracture	Low	< 2.5	0.8
	Medium	2.5–7.5	1
	High	> 7.5	1.15
Hip fracture	Low	< 2.5	0.65
	Medium	2.5–7.5	1
	High	> 7.5	1.2

*Use correcting factor to multiply the 10-year probability of fracture calculated from the FRAX tool for the final result in patients treated with glucocorticoids. Data from Kanis et al. 2011 [22]

## FRAX adjustment for aromatase inhibitors and androgen depletion therapy

In its original form, FRAX has not been designed to assess fracture risk in women with breast cancer, or men with prostate cancer, or to assess in any other form of cancer for both men and women. Suggestions were raised to include cancer under the “secondary osteoporosis” option. However, the secondary osteoporosis input only affects FRAX calculations when BMD is not entered, but not when BMD is included. This was based on the assumption that the risk would be mediated through BMD [34]. Consequently, this may substantially underestimate the effect of cancer on the fracture risk assessment particularly in women with breast cancer treated with aromatase inhibitor (AI) or men with prostate cancer receiving androgen deprivation therapy (ADT); both therapies are known for their negative impact on bone health. Also, the “secondary osteoporosis” option in the FRAX tool has a much smaller effect on fracture risk than would be expected for women or men treated with these therapies. Interestingly, comparing AIs with tamoxifen mature, it was evident that AI have a large effect on acute fracture risk during active treatment [45, 46]. This may be underestimated by FRAX, as the algorithm designed to provide long-term (10 years) fracture risk. More recent data have rated the independent fracture risk in aromatase inhibitor bone loss or androgen deprivation therapy as equivalent to that seen in rheumatoid arthritis. As a result, it has been suggested recently to use the bypass of rheumatoid arthritis in FRAX as it has been proposed in type 2 diabetes [47].

## FRAX adjustment for type II diabetes mellitus

Although patients with type II diabetes mellitus have higher BMD measures [48], this was reported to be associated with an increased risk of osteoporotic fractures, independently of FRAX probability [49]. Four proposed methods to improve the performance of FRAX for type II diabetes mellitus were compared by using the Manitoba BMD Registry data: by including the rheumatoid arthritis (RA) input to FRAX; making a trabecular bone score (TBS) adjustment to FRAX; reducing the femoral neck T-score input to FRAX by 0.5 SD; and increasing the age input to FRAX by 10 years [50]. This found that diabetes was associated with increased risk for major osteoporosis fracture and hip fractures, over a mean of 8.3 years. Unadjusted FRAX risk in patients with type II diabetes underestimated major osteoporosis fracture (observed/predicted ratio 1.15; 95% CI 1.03–1.28); however, after applying the diabetes adjustments, this was no longer significant. In concordance, hip fracture risk was more severely underestimated (observed/predicted ratio 1.85; 95% CI 1.51–2.20) being only partially corrected by applying the diabetes adjustments (still significant for the RA and TBS adjustments). Therefore, whilst FRAX underestimates the fracture risk assessment in patients with type II diabetes mellitus, applying all these strategies was found to improve fracture prediction. However, no single method was optimal in all settings.

## FRAX adjustment to incorporate falls history

For several reasons, FRAX algorithm did not incorporate falls history. There was some doubt among FRAX developers that characterising risk on this basis may identify a group amenable to therapeutic intervention. This arose mainly from the risedronate hip trial (Hip Intervention Program Study Group) [51], as this revealed that risedronate significantly reduces the risk of hip fracture among elderly women who had confirmed osteoporosis but not, however, among those selected primarily on the basis of risk factors other than low BMD. In contrast, a clodronate study found that treatment works in fallers compared with non-fallers [52]. Also, a recent study revealed that denosumab has double positive beneficial effect not only BMD but also on the reduction of falls risk [53]. Considering other factors included in FRAX model, such as age and parental history of hip fracture, these are not amenable to bone-directed interventions; therefore, it is not logical for falls to be excluded on this basis. Also, falls history was only documented in only a minority of cohorts at the time of the development of FRAX algorithm. Out of the 12 original cohorts, only three (25%) had information regarding falls. As there was not the breadth of data compared with the other risk variables, this limited the ability to look for association between falls and the other risk variables. When establishing the dataset leading to the FRAX algorithm, there was variation in the construction of the question between cohorts (Have you fallen in the past week?/in the past month?/in the last 6 months?/in the last year?). As a result, the prevalence of falls varied markedly, more by question construct than by age. Lastly, although falls were associated with a significant increase in fracture; this was reported in some, but not all, cohorts used in the FRAX development. Subsequently, there was heterogeneity in the outcome, possibly related to the heterogeneity of the construct of the question [30].

In efforts to incorporate falls risk into the FRAX algorithm, the FRAX-Falls Clinical Task Force Sub-Committee suggested different options were suggested [30]. These included the following: adding the number of falls in the previous year as a separate risk factor to the FRAX algorithm; make the FRAX user aware of the current limitations of FRAX (i.e. lack of falls history as a risk factor); incorporating other parameters such as sarcopenia, frailty, and functional status in FRAX trying to further improve 10-year fracture risk assessment; or to add guidance to clinicians in FRAX by including statements about the importance of falls prevention. Whilst incorporation of the falls risk or the number of falls as a separate risk factor seems to be the more likely practical option, an alternative could be combined assessment of the individual patient’s fracture as well as falls risk [54].

## FRAX to re-think

There have been several different risk assessment tools that have been developed; however, only six tools were validated in a population-based setting with a proper methodological quality. These include 3 tools developed to predict low BMD (or the need for a BMD): OST, ORAI, and SCORE, whereas the other 3 tools were developed aiming at fracture prediction: Garvan, Q Fracture, and FRAX. As noted by the authors [55], the utility of a tool relies not only on its diagnostic accuracy but also on its ease of use and ability to achieve its expected targets [56]. Ideally, risk factors should be collected through patient self-report and need to be unambiguous as well as easily determined. Earlier data revealed that in predicting fractures, simpler models or tools perform as well as the more complex ones [57]. As each tool has its unique strengths and weaknesses, the aim is to monitor each individual scoring system’s performance in order to develop create better tools, with better effective screening strategies, eventually improving the patients’ care worldwide.

If limited to just one or 2 clinical risk elements, modification or adjustment of the FRAX risk assessment score could be a good option in overcoming the underestimation of the absolute fracture risk. However, so far up to 10 adjustments have been identified, to improve the performance of the FRAX prediction tool, which is too many and as a result has left the current FRAX patchy. Due to this extra burden has been thrown on to the treating clinicians who are trying to find the best approach for their patients in standard clinical practice. This is supported by earlier reports revealing that primary care physicians embrace the concept of absolute fracture probability [58], which instantaneously reduced their tendency to initiate treatment of osteopenia [59]. Furthermore, primary care physicians were, still, found to be reluctant to treat women identified as being at high risk of fracture in the absence of a BMD T-score in the osteoporotic range [60]. Even after celebrating the 10th FRAX birthday, it remains a challenge for osteoporosis community to move beyond BMD T-scores.

Over the last 3 years, major developments in fracture risk assessment have been the publication of three large pragmatic clinical trials from community-based screening, the Screening for Osteoporosis in Older Women for the Prevention of Fracture (SCOOP) [61], Risk-Stratified Osteoporosis Strategy Evaluation Study (ROSE) [62], and SALT Osteoporosis Study (SOS) [63] trials. However, in the 3 studies, there is no evidence that FRAX use reduces osteoporotic fractures, except hip, and all the 3 studies failed to show a statistically significant result on primary outcomes. In the SCOOP trial [61], at the 10th percentile of baseline FRAX hip probability, hip fractures were not significantly reduced in the intervention vs. control groups, whereas at the 90th percentile, the intervention group (vs. control group) experienced a 33% reduction in hip fractures. Similarly, in ROSE trial [62], by intention to treat, there was no difference in the primary outcome (osteoporosis-related fractures) after median follow-up of 5 years. Lastly, in SOS trial [63], after a mean follow-up of 3.7 years, the intention to treat analysis showed no statistically significant effect on the primary fracture outcome (HR 0.97; 95% CI 0.87–1.08) nor on secondary outcomes (osteoporotic fractures HR 0.91; 95% CI = 0.81–1.03, major osteoporosis fracture HR 0.91; 95% CI 0.80–1.04, hip fractures HR 0.91; 95% CI = 0.71–1.15). This pattern of less accurate fracture reduction, particularly of non-hip fractures, is supported by the lack of provision for lumbar spine BMD which is commonly recommended in treatment guidelines, meaning that FRAX does not account for individuals who have low lumbar T-score but with normal femoral neck. This comes in concordance with published data revealing that in contrast to osteoporotic vertebral fractures, hip fractures represent only a small portion of osteoporotic fractures (14%) [64]. These findings clearly illustrate the vital need for re-thinking the FRAX algorithm.

Strategies are desperately needed to improve targeting and effectiveness of the screening programs, particularly among younger individuals where prediction tools have not performed well [65, 66]. The emerging strong data revealing the importance of specific risk factors, such as previous falls, type II diabetes mellitus, and imminent fracture risk, pave the way for the suggestion to incorporate additional risk factors in the next generation of fracture prediction tools. However, such prediction algorithms require independent external validation before being adopted in standard clinical practice.

To improve the identification of individuals at high risk much earlier, the next challenge will be incorporating these advancements to our everyday work-flows. Adopting this change would impact on the ongoing current crisis in osteoporosis management. There is currently speculation regarding the development of a new FRAX tool; however, so far, it is not clear to what extent a second generation of FRAX would be able to address those limitations perceived with the current FRAX prediction tool. There is a long wish list for change, as traditionally expected, with compromises being made along the way, and this will perhaps leave some disappointed.

## A glimpse at the future

In recent years, there has been a rapid expansion in the development and use of digital technologies. These advances have had a positive global impact, ranging from robotics, wearable health devices, and artificial intelligence (AI). Inspired by the human brain functioning processes, in particular the adaptation to solve non-linear problems and to discover subtle trends and associations among variables, AI are computational adaptive systems which have been developed mimicking this thinking approach [67, 68]. Over the past few years, AI proved itself to be valuable in understanding and linking the relations between variables of complex systems such as those reported in multifactorial osteoporosis. Both machine learning and deep learning models have found applications in osteoporosis. Several studies have been published with the aim of either to predict an indicator of osteoporosis, such as BMD or fractures, or as a tool for automatic segmentation of the images of patients with or at risk of osteoporosis. Examples are those tools which used the supervised category of models to predict categorical outcomes, specifically the fractures/no-fracture classes [69–71] and osteonecrosis [72], whereas others predict quantitatively the BMD value [73, 74].

In conclusion, the use of fracture assessment tools is imperative in moving forward to close the large care gap. Targeting individuals with increased risk of osteoporotic fracture is an important challenge in the field of osteoporosis. Out of all the absolute fracture risk prediction tools, FRAX still keeps its place as the most commonly used program in standard clinical practice. By identifying which patients would benefit most from DXA scanning or treatment, risk assessment tools may contribute to health care decision-making. As time passes, and after 10 years of using FRAX on a wide scale, teething problems started to appear which required further adjustments of the original FRAX. However, as the adjustment list got longer, making it difficult to keep patching over the original FRAX, there have been calls for a newer version of FRAX which would be able to address such perceived limitations. Alternatively, a newer sophisticated algorithm integrating not only multiple risk factors such FRAX variables but also advanced imaging parameters, physical performance measures, and genetic data can be developed. Though still in development, recent developments in artificial intelligence have had a successful application in aid of osteoporosis diagnosis and fracture risk assessment.
